# Reflections on year one of a new residency program: Lessons for future leaders from residents and educators

**DOI:** 10.15694/mep.2020.000094.1

**Published:** 2020-05-08

**Authors:** Benjamin L Moresco, Jesse Banales, Megan Harline, Amanda Phan, Danielle Ramirez, Lauren Sadovsky, Diana Villarreal, Michelle D Barajaz, Sarah F Denniston, Adam Wolfe

**Affiliations:** 1 Massachusetts General Hospital; 2 University of Texas - Southwestern; 3 Partners in Pediatrics; 4 Northwestern University Feinberg School of Medicine; 5 Mednax Medical Group of Texas; 6 Cevey Pediatrics; 7 Loma Linda University; 8 Baylor College of Medicine - The Children's Hospital of San Antonio; 9 Tufts University

**Keywords:** New program, recruitment, graduate medical education, resident perspective, chief resident, senior resident

## Abstract

This article was migrated. The article was marked as recommended.

As the number of graduating medical students increases, the number of primary care residency positions is not keeping pace. One solution to this problem is the creation and accreditation of new residencies, although there is little literature describing the perspectives of the residents and educators who found new programs. Seven out of ten resident physicians who served as the inaugural interns in a new residency training program in pediatrics provide their reflection on the distinctive perspective they had from this experience. They have identified consensus themes in topic areas of strengths, challenges, and lessons learned from training in a new program. Themes applying to strengths of participating in a new residency training program were the opportunity to shape the program, individualized learning experience, and enthusiastic faculty. Challenges of a new program included missing upper level residents, diverse faculty expectations, and morale. Themes under lessons learned included resident engagement, expectations and feedback, and wellness. Each theme was then considered in the context of the medical education literature, underscoring the important balance that new program leaders must strike between structure and flexibility. This inaugural resident class has identified key challenges and opportunities to inform education leaders who are planning new GME training programs.

## Introduction

The graduate medical education (GME) community in the United States faces a daunting challenge: the Association of American Medical Colleges (AAMC) has repeatedly predicted a significant physician shortage, and thus an increasing enrollment of medical students (Dall *et al.*, 2016). Unfortunately, this change has been occurring without available residency positions increasing accordingly (Dall *et al.*, 2016). One approach proposed to meet this need is the creation and accreditation of new residency training programs ( Barajaz and Turner, 2016).

The Children’s Hospital of San Antonio, in partnership with Baylor College of Medicine, developed a new categorical pediatrics residency training program, started recruitment, and began training our first class of ten interns in July 2015. The hospital itself was also new, with more than 150 new faculty members in pediatrics hired from across the United States and internationally. The residency program’s vision was to engage learners with an unprecedented amount of learner-driven customizability and a strong emphasis on resident feedback for continuous quality improvement in the educational experience. During the first year of recruitment, prospective residents were informed that 18 of the 39 program training rotations were customizable to individualized learning goals, and that they would work one-on-one with faculty to develop these goals given the absence of senior residents.

Following completion of their first training year in July of 2016, members of our inaugural intern class gathered with program leadership to reflect on the successes and challenges of this experience. We present here a distinctive perspective, crafted by learners and with reflection by educators, on the experience of training in a new program at a new hospital. The lessons learned can guide future educators seeking to build new residency training opportunities.

## Perspectives of the inaugural residents

We focused recruitment of the first class of ten residents on candidates with enthusiasm to help build the program who would participate in program feedback and serve as ambassadors for future recruitment. Six members of our first class of interns matched from in-state medical schools (Texas), with four others coming from Missouri, Nebraska, Rhode Island, and Washington state. All ten matched from within our top 20% of ranked applicants.

Seven residents (7/10; 70% of the class) prepared personal responses to six open-ended questions: (1) What qualities, features, or opportunities made you choose to rank a newly-formed residency program for your training? (2) Following this first year, what do you think has been the greatest strength of training in a new residency program and how has that impacted your training for your future career? (3) Following this first year, what do you think has been the greatest challenge you have faced as a direct result of being in a new training program? (4) How did you cope with or manage the challenge you described in question #3? (5) What advice would you give to residents who are about to join a new or expanding training program? (6) What advice would you give to those leading a new residency program for ways to support their first-year trainees and maximize the educational impact?

We individually conducted a keywords-in-context analysis of the pooled, anonymized responses ( Leech and Onwuegbuzie, 2007), then met as a group to identify consensus themes in each of three topic areas: strengths of participating in a new program, challenges of a new program, and lessons learned. The resident authors then selected text from the original responses that best illustrated each of the themes. We assembled these into a single narrative, which was group edited on a shared on-line document by all seven resident authors. Here we describe the residents’ consensus themes, followed by a discussion from program leaders placing the resident perspectives in the context of the literature.

## Strengths of participating in a new residency training program

*Opportunity to shape the program*The chance to meaningfully influence the development of the residency was the most important draw to our new program. This potential influence made the program stand out from a large pool of other well-established training institutions. Given prior work experiences in rigid systems resistant to change, the opportunity to shape the culture of learning appealed to us. As interns, we felt a greater sense of purpose by not simply maintaining the function of an established system, but instead by helping to create a residency that will impact future generations of pediatric residents. As trainees, we were encouraged to give direct and frequent feedback for a system we were helping create. *Individualized learning experience*The unusual ability of a residency program to offer an individualized learning experience greatly appealed to us. This was an important factor when deciding to rank the program: a personalized curriculum and a focus on individualized resident education and development. Since there were no upper-level residents, we were given the opportunity to rise above the training of an intern. We had more responsibilities and more opportunities for procedures. Through direct interaction with faculty and staff, we identified individual educational needs and clinical teaching felt truly personalized. This fostered an environment of ownership and responsibility for our own training, leading to improved leadership skills. Overall, we perceived the program to have increased attention to our success via encouragement to provide feedback and partake in an educational experience with our specific needs and goals as the focus. *Enthusiastic and available faculty* Energetic and passionate faculty helped us feel genuinely welcomed and supported during both the recruiting season and intern year. As they were recently recruited themselves to join a new children’s hospital, faculty members were invested in teaching and in encouraging our successes as individuals. With the capability to work one-on-one with attending physicians, we had unfiltered access to their clinical expertise and wisdom while also benefiting from numerous career opportunities and research mentorships. This dedicated group of faculty gave us more confidence in our decision to invest in a new residency training program.

## Challenges of a new program

*Lack of upper level residents* An immediate challenge we faced was not having senior residents. Senior residents can directly shape the learning environment and offer much needed guidance to interns. Interns look to upper levels to model their position and establish role identity and expectations. Many of us found that it was difficult to gauge the normalcy of what we were experiencing because there were no upper level residents to guide our perspective. Interns learn much from the residents who are 1-2 years ahead of them in training, and their absence poses a distinct challenge for a new training program.

*Inconsistent faculty expectations* We quickly realized that faculty expectations of us varied widely, from unrealistically high expectations to disappointingly low expectations. It seemed that some attending physicians assumed that we were the “best in the country.” Other attending physicians appeared to assume that we should act as senior-level residents rather than as interns. On the other end of the spectrum, some attending physicians created the impression that they preferred for us to merely observe patient care, with minimal autonomy. Some services may have found it difficult to incorporate an intern into their already established routine, especially those that relied heavily on advanced practice professionals.

*Resident morale* The challenge of sustaining an optimistic attitude while facing a seemingly experimental year of training was a common theme among the new class. We each faced times during our first year when the appeal of starting a new program was challenged by the reality of the growing pains that accompanied it. Morale declined as we realized that we had less of a voice to instill change than we first anticipated. This was compounded by the unknown of whether the program would be successful. Feelings of loneliness arose, which was thought to be related to a lack of upper level and peer support on individual rotations.

*Coping with challenges* Coping with the challenges of the new program requires a unique set of qualities of those recruited. A strong sense of self-motivation drove us to be proactive, rather than passive, in the program. Many of us sought opportunities to augment our clinical education such as proposing quality improvement projects, promoting program development, conducting independent research, and participating in national meetings. Maintenance of good communication, whether it was direct through attending feedback or indirect through our quarterly Voice in the Program sessions (where we identified strengths, weaknesses, opportunities, and threats for each component of the training environment) also proved to be crucial in addressing the challenges.

## Lessons learned

### Resident engagement

Residents who are proactive in pursuing educational goals and those who are more passively guided by their program can all be successful in residency and become competent physicians. However, in the context of training in a new program, we feel that more proactive residents may be more successful. They can contribute the most to a developing program by actively participating in patient care, contributing to scholarship, and crafting smart goals to effect change. They also tend to be open-minded and can think critically through problems and compromise on solutions. The residents who fit best with our new residency program were patient, mindful of the growing pains, and willing to constantly make changes to improve the efficiency and experience of everyone involved.

### Standard expectations and feedback

The goals of a new residency program should include standardized and realistic expectations, open lines of communication, and an environment agreeable to change. A new program should provide a clearly defined set of expectations for each rotation agreed upon by all rotation faculty to guide the interns toward the learning objectives expected to be achieved by the end of the rotation. A program should also provide a framework that frequently solicits feedback from the residents and be prepared to implement changes quickly. Feedback can be accomplished with monthly resident feedback meetings, open-door policies, leaders who demonstrate empathy, and frequent updates on prior concerns or ideas brought up by residents. Residents should be flexible, creative, and not afraid to speak up with novel ideas or ways to improve the system. Everyone should be able to give and receive feedback regardless of level of training. A mutual respect as well as a unified effort for excellence must exist between the program faculty and residents for a new program to thrive. All members of the team need to be heard and know that their contribution is valued.

### Resident-driven wellness

We encourage program leaders to implement a resident-run support system that readily integrates incoming residents, requires little maintenance, and requires little administrative effort. We organized three opportunities for resident-run support: housestaff meetings, fireside chats, and wellness/social events. Housestaff meetings allow the residents to regroup and communicate about national meetings, child advocacy opportunities, ongoing research and quality improvement, and hospital initiatives. Fireside chats provide an informal way to debrief in a safe zone amongst co-residents. These chats exclude program leaders and involve a chief resident to mediate the discussion. It is helpful to have a chief resident who is an advocate for the interns. The effective chief resident listens to resident concerns, assists in brainstorming creative solutions to problems, and provides encouragement and an uplifting perspective. In addition to structured housestaff meetings and fireside chats, the emphasis on wellness should also be resident run. One of the challenges we faced in integrating wellness into the curriculum was the perception that there is no room or time; however, opportunities for residents to check in with each other in a residency-sponsored event may be one of the most important aspects of preventing burnout, enhancing resident camaraderie, and eliminating feelings of isolation. Besides camaraderie, residents need to see their leadership caring about them, reassuring them, and guiding them in their development as future physicians.

### Set appropriate expectations for residents and faculty

Reciprocal enthusiasm and engagement from the teaching faculty was key to this class’ recruitment and to their morale. We saw significant benefit from one-on-one clinical mentorship from faculty throughout the first year, especially in the absence of senior residents. At the same time, faculty expectations of the first intern class need to be set appropriately and consistently so that we do not forget that clinical responsibilities of interns are different from those of senior residents.

## Discussion

This resident class was recruited in part based on their pioneering spirit and enthusiasm to help build a new program. Despite the importance of developing new GME training opportunities, there is limited literature addressing the challenges of creating and recruiting residents to new training programs ( Barajaz and Turner, 2016; Barajaz *et al.*, 2018). The themes identified by our residents have provided essential insight into the experience of learners in a new residency.

The unusual opportunity to help build a new program as they trained was clearly attractive to this first class, but it also yielded unusual challenges. Our first resident class shouldered an immense emotional responsibility for the success of this program. They held themselves to an unrealistic expectation that they should feel responsible for any failures or challenges we faced, and that the perceived slow progress of change within the hospital was directly attributable to them. They may have been unnecessarily distracted and dismayed by the slow pace of global change in the hospital setting, which is not unique to new programs. As a result, they may not have appreciated the significant impact that they have had on the growth and progress of the educational, clinical, and scholarly enterprises of the hospital. As they identified, the inaugural residents need to have clear expectations of how they are expected to help shape the program in addition to a realistic expected tempo of change.

Senior residents play an essential role in many aspects of resident training, including identification of systems-based practice issues ( Li *et al.*, 2015), upholding expectations and standards ( Cho *et al.*, 2014), supervision and “near-peer” feedback ( Petrilli, Del Valle and Chopra, 2016; Tully *et al.*, 2016), and generation of perceptions of the educational experience ( Haferbecker *et al.*, 2013; Rangachari *et al.*, 2017). Seniors are also essential to the training of handoffs and transitions of care along with quality and patient safety ( Date *et al.*, 2013; Van Leer *et al.*, 2015; Huda, Faden and Goldszmidt, 2017; Kimura *et al.*, 2018) and defining the resident as teacher ( Fisher, 2016; Raty *et al.*, 2017). Senior residents have further been found to be important for resident wellness in setting expectations of workload, differing social relationships, social pressures, and mutual trust development that leads to the productive clinical learning environment within a new hospital, curriculum, and residency ( Tsai *et al.*, 2014; Kimura *et al.*, 2018).

In hindsight, it likely would have proven helpful to describe the roles of senior residents to this intern class and our faculty early on, so residents would feel more empowered to ask for help from faculty or from their chief resident that they might otherwise have been more comfortable addressing with senior peers. We partially corrected this during October of the first year by holding a workshop entitled, “What your senior residents would tell you if you had senior residents.” This session, led by the chief resident and junior faculty, helped to provide context for the challenges that arose from lack of upper level trainees.

A strong chief resident may be able - in some ways - to serve as a surrogate for the key roles of senior resident. The chief may also have a myriad educational and administrative responsibilities ( Parrino and Steel, 1983; Warner *et al.*, 2007; Lim *et al.*, 2009; Dabrow *et al.*, 2011; McPhillips *et al.*, 2011; Deane and Ringdahl, 2012), which should be well defined early by the program director in order to provide a foundation on which to build. In a new program, the chief resident also should have an expectation of providing a substantial degree of counseling and advising. This role has been previously described as a “reluctant staff therapist” ( Green, 1975; Gil *et al.*, 2009), and the right individual for a new program should have an appropriate skillset and embrace this role. The chief resident - presumably recruited from another site or program - would also likely be maneuvering through a very complicated and tumultuous year in a new hospital system. They should be effectively and proactively trained to avoid the pitfalls associated with middle management, such as appearing to “side up” with program leadership or “side down” with residents ( Berg and Huot, 2007; Hafner *et al.*, 2010). The chief resident may also need to focus early on teaching residents how to be leaders ( Parrino and Steel, 1983; Biese *et al.*, 2011) and teachers ( Fisher, 2016; Raty *et al.*, 2017). Well-established roles and expectations, along with training prior to the start of the chief residency year, lead to success, comfort, and improvement of relationships experienced by both the program director and chief resident ( Warner *et al.*, 2007; Lim *et al.*, 2009; Dabrow *et al.*, 2011; McPhillips *et al.*, 2011; Deane and Ringdahl, 2012).

In the absence of senior residents, our inaugural interns also sought senior peer guidance from faculty. For future new programs, we would suggest specific preparation of the faculty in educating the first class, emphasizing the importance of direct supervision while still offering autonomy in patient care as appropriate, especially surrounding handoffs and transitions of care ( Date *et al.*, 2013; Huda, Faden and Goldszmidt, 2017; Raty *et al.*, 2017). This could be established with faculty development efforts to engage all teaching faculty before the residents start their residency. Providing graded autonomy on inpatient services has been shown not to be detrimental to quality of care or patient safety ( Van Leer *et al.*, 2015), and based on our residents’ reflections, should positively impact the learner experience.

Finally, this inaugural class reminded us that residents are the prime stakeholders in their own wellness. As they experienced the stresses of being an only class, lacking senior peers to encourage them, the class came together to build a culture of wellness rooted in engagement with our new residency. They showed us how intertwined the concepts of wellness and morale can be ( Tsai *et al.*, 2014; Kimura *et al.*, 2018). The development of their interactions with their chief resident, structured feedback sessions to program leadership, and peer-supported wellness activities was driven by residents. This culture continues now as this first class has graduated from residency and has indelibly empowered future classes to continue to engage with matters of morale and well-being.

## Conclusions

Our program’s first intern class felt challenged to balance a need for standardization and structure versus a desire for flexibility and continuous improvement. They exhibited engagement and enthusiasm to build the program collaboratively with leadership, while struggling with the lack of peer leaders and feeling a lack of consistently-applied expectations. Some sacrifice of clear and secure boundaries allowed for rapid improvements within the program and maximized resident input from year one. Striking this balance between structure and flexibility may be the key to success for both learners and leaders in the development of new GME programs.

## Take Home Messages

• New residency programs are needed to meet projected increasing physician demand; little is known about how to do this successfully
• First year residents from a new pediatrics training program present their perspectives on their experience
• Strengths of a new program include opportunity to shape the program, individualized learning experience, and enthusiastic faculty
• Challenges of a new program include lack of upper level residents, inconsistent faculty expectations, and threats to morale
• New program leaders should include lessons learned to recruit residents with the best chance of success, including emphasizing their proactivity, and balancing the importance of structure and flexibility as a new program develops

## Notes On Contributors

**Benjamin L. Moresco**, MD is a clinical fellow in pediatric hospice and palliative care medicine at Massachusetts General Hospital, Boston, MA; he was an inaugural intern in our program and served as our first graduate chief resident in 2018-2019. ORCiD: https://orcid.org/0000-0003-3492-1038.

**Jesse Banales**, MD is a clinical fellow in neonatal-perinatal medicine at University of Texas - Southwestern, Dallas, TX. He was an inaugural intern in our program.

**Megan Harline**, MD is a general pediatrician at Partners in Pediatrics practicing in Denver, CO. She was an inaugural intern in our program.

**Amanda Phan**, MD is an Instructor of Pediatrics at Northwestern University Feinberg School of Medicine in Chicago, IL. She was an inaugural intern in our program.

**Danielle Ramirez**, MD is a general pediatrician at the Pediatrix Medical Group of Texas practicing in San Antonio, TX. She was an inaugural intern in our program.

**Lauren Sadovsky**, DO is a general pediatrician at Cevey Pediatrics practicing in San Antonio, TX. She was an inaugural intern in our program.

**Diana Villarreal**, MD, PhD is an assistant professor of pediatrics at Loma Linda University practicing in Loma Linda, CA. She was an inaugural intern in our program.

**Michelle D. Barajaz**, MD is a general pediatrician and founding Program Director of the Pediatric Residency Program at Baylor College of Medicine - The Children’s Hospital of San Antonio; San Antonio, TX. ORCID: https://orcid.org/0000-0002-3986-088X.

**Sarah F. Denniston**, MD is an Assistant Professor of Pediatrics and Director of the Pediatric Hospital Medicine Fellowship at Tufts University, Boston, MA. She served as a founding Associate Program Director of our program.

**Adam D. Wolfe** is an Assistant Professor of Pediatrics, Associate Director of the Pediatric Residency Program, and Assistant Dean of Education at Baylor College of Medicine - The Children’s Hospital of San Antonio, San Antonio, TX. ORCiD: https://orcid.org/0000-0002-3113-2298.

## Declarations

The author has declared that there are no conflicts of interest.

## Ethics Statement

This manuscript includes the collected perspectives of the authors, and does not involve any investigation of human subjects.

## External Funding

This article has not had any External Funding

## References

[ref1] Barajaz M. Kumar S. Denniston S. F. and Wolfe A. D. (2018) If You Build It, Will They Come? An Analysis of Candidate Attitudes Toward A New Residency Program, MedEdPublish, 10.15694/mep.2018.0000245.1 PMC10711978 38089192

[ref2] Barajaz M. and Turner T. (2016) Starting a new residency program: a step-by-step guide for institutions, hospitals, and program directors. Med Educ Online. 21, p.32271. 10.3402/meo.v21.32271 27507541 PMC4978854

[ref3] Berg D. N. and Huot S. J. (2007) Middle manager role of the chief medical resident: an organizational psychologist’s perspective. J Gen Intern Med. 22(12), pp.1771–1774. 10.1007/s11606-007-0425-8 17940827 PMC2219837

[ref4] Biese K. Leacock B. W. Osmond C. R. and Hobgood C. D. (2011) Engaging senior residents as leaders: a novel structure for multiple chief roles. J Grad Med Educ. 3(2), pp.236–238. 10.4300/JGME-D-10-00045.1 22655148 PMC3184899

[ref5] Cho C. S. Delgado E. M. Barg F. K. and Posner J. C. (2014) Resident perspectives on professionalism lack common consensus. Ann Emerg Med. 63(1), pp.61–67. 10.1016/j.annemergmed.2013.07.493 23948747

[ref6] Dabrow S. M. Harris E. J. Maldonado L. A. and Gereige R. S. (2011) Two perspectives on the educational and administrative roles of the pediatric chief resident. J Grad Med Educ. 3(1), pp.17–20. 10.4300/JGME-D-10-00039.1 22379517 PMC3186279

[ref7] Dall T. West T. Chakrabarti R. and Iacobucci W. (2016) The Complexities of Physician Supply and Demand 2016 Update: Projections from 2014 to 2025. Prepared for the Association of American Medical Colleges.Available at: https://www.modernhealthcare.com/assets/pdf/CH10888123.PDF(Accessed: 08/19/2019).

[ref8] Date D. F. Sanfey H. Mellinger J. and Dunnington G. (2013) Handoffs in general surgery residency, an observation of intern and senior residents. Am J Surg. 206(5), pp.693–7. 10.1016/j.amjsurg.2013.07.006 24035213

[ref9] Deane K. and Ringdahl E. (2012) The family medicine chief resident: a national survey of leadership development. Fam Med. 44(2), pp.117–120. https://www.ncbi.nlm.nih.gov/pubmed/22328478 22328478

[ref10] Fisher J. F. (2016) Permanent resident. Med Educ Online. 21, p.31160. 10.3402/meo.v21.31160 27193992 PMC4871894

[ref11] Gil K. M. Savitski J. L. Bazan S. Patterson L. R. (2009) Obstetrics and gynaecology chief resident attitudes toward teaching junior residents under normal working conditions. Med Educ. 43(9), pp.907–911. 10.1111/j.1365-2923.2009.03422.x 19709015

[ref12] Green S. A. (1975) The chief resident as reluctant staff therapist. Am J Psychiatry. 132(10), pp.1078–1081. 10.1176/ajp.132.10.1078 1166882

[ref13] Haferbecker D. Fakeye O. Medina S. P. and Fieldston E. S. (2013) Perceptions of educational experience and inpatient workload among pediatric residents. Hosp Pediatr. 3(3), pp.276–84. 10.1542/hpeds.2012-0068 24313098

[ref14] Hafner J. W. Gardner J. C. Boston W. S. and Aldag J. C. (2010) The chief resident role in emergency medicine residency programs. West J Emerg Med. 11(2), pp.120–125. https://www.ncbi.nlm.nih.gov/pubmed/20823957 20823957 PMC2908642

[ref15] Huda N. Faden L. and Goldszmidt M. (2017) Entrustment of the on-call senior medical resident role: implications for patient safety and collective care. BMC Med Educ. 17(1), p.121. 10.1186/s12909-017-0959-3 28705161 PMC5513049

[ref16] Kimura T. Seo E. Ogawa R. Matsumura S. (2018) Recovery from a depressive episode during postgraduate residency training is associated with senior doctors’ support. J Gen Fam Med. 19(1), pp.20–26. 10.1002/jgf2.144 29340262 PMC5763017

[ref17] Leech N. and Onwuegbuzie A. (2007) An array of qualitative data analysis tools: A call for data analysis triangulation. School Psychology Quarterly. 22(4), pp.557–584. 10.1037/1045-3830.22.4.557

[ref18] Li S. T. Paterniti D. A. Tancredi D. J. Burke A. E. (2015) Resident Self-Assessment and Learning Goal Development: Evaluation of Resident-Reported Competence and Future Goals. Acad Pediatr. 15(4), pp.367–373. 10.1016/j.acap.2015.01.001 26142068

[ref19] Lim R. F. Schwartz E. Servis M. Cox P. D. (2009) The chief resident in psychiatry: roles and responsibilities. Acad Psychiatry. 33(1), pp.56–59. 10.1176/appi.ap.33.1.56 19349446

[ref20] McPhillips H. A. Frohna J. G. Murad M. H. Batra M. (2011) Enhancing teamwork between chief residents and residency program directors: description and outcomes of an experiential workshop. J Grad Med Educ. 3(4), pp.593–597. 10.4300/JGME-D-10-00226.1 23205220 PMC3244337

[ref21] Parrino T. A. and Steel K. (1983) The medical chief residency and its relation to academic sections of general medicine. Am J Med. 75(5), pp.839–842. 10.1016/0002-9343(83)90415-1 6638050

[ref22] Petrilli C. M. Del Valle J. and Chopra V. (2016) Why July Matters. Acad Med. 91(7), pp.910–912. 10.1097/ACM.0000000000001196 27119323

[ref23] Rangachari D. Brown L. E. Kern D. E. and Melia M. T. (2017) Clinical coaching: Evolving the apprenticeship model for modern housestaff. Med Teach. 39(7), pp.780–782. 10.1080/0142159X.2016.1270425 28024461

[ref24] Raty S. R. Teal C. R. Nelson E. A. and Gill A. C. (2017) Near-peers improve patient safety training in the preclinical curriculum. Med Educ Online. 22(1), p.1289315. 10.1080/10872981.2017.1289315 28219315 PMC5328309

[ref25] Tsai J. C. Chen C. S. Sun I. F. Liu K. M. (2014) Clinical learning environment measurement for medical trainees at transitions: relations with socio-cultural factors and mental distress. BMC Med Educ. 14, p.226. 10.1186/1472-6920-14-226 25335528 PMC4287428

[ref26] Tully K. Keller J. Blatt B. and Greenberg L. (2016) Observing and Giving Feedback to Novice PGY-1s. South Med J. 109(5), pp.320–325. 10.14423/SMJ.0000000000000459 27135733

[ref27] Van Leer P. E. Lavine E. K. Rabrich J. S. Wiener D. E. (2015) Resident Supervision and Patient Safety: Do Different Levels of Resident Supervision Affect the Rate of Morbidity and Mortality Cases? J Emerg Med. 49(6), pp.944–948. 10.1016/j.jemermed.2015.05.033 26234717

[ref28] Warner C. H. Rachal J. Breitbach J. Higgins M. (2007) Current perspectives on chief residents in psychiatry. Acad Psychiatry. 31(4), pp.270–276. 10.1176/appi.ap.31.4.270 17626188

