# Supplementary material for: Reflections on year one of a new residency program: Lessons for future leaders from residents and educators
Source: MedEdPublish (2016). 2020 May 8;9:94. [Version 1] doi: 10.15694/mep.2020.000094.1 (PMC10697504; doi:10.15694/mep.2020.000094.1)
Supplement: Peer review history

# Reviewer report: Reviewer response for version 1

**DOI:** 10.21956/mep.20019.r30546

**Published:** 2020-05-25

**Authors:** Veena Rodrigues

**Affiliations:** 1 Norwich Medical School

This review has been migrated. The reviewer awarded 3 stars out of 5 Interesting paper focusing on a new residency program in USA. Several strengths such as the opportunity to shape the program, and create a personalized learning experience were highlighted; challenges faced included the morale of the residents as well as differing faculty expectations. The lessons learned are likely applicable to the setting up of any new, postgraduate, medical training programs.In my view, issues that would have strengthened this paper are:1) A discussion of the supervision model used within this training program: While it is clear that there were no senior residents to provide feedback, mentoring and support, this was known prior to launching the program. Arrangements could have been made for other supportive measures to be put in place proactively, for example, one to one or group supervision by the chief resident or junior faculty, creation of action learning sets, etc. Individual residents can take responsibility for morale and wellness but surely at least some of this responsibility lies with the program too? Good supervision needs to be a balance between the formative, normative and restorative elements of supervision.2) The authors say, “Faculty expectations of the first intern class need to be set appropriately and consistently so that we do not forget that clinical responsibilities of interns are different from those of senior residents”. I would have liked to see a fuller discussion of the expectations of the program from the residents versus the expectations of the residents from the program, especially the extent of any potential mismatch between the two.3) A discussion of specific changes made to this residency program in view of the lessons learned - what changes have been put in place based on the authors’ recommendations so that the identified challenges could be minimized for future residents joining this program?In summary, this paper highlights the importance of careful planning prior to setting up a new residency training program for mutual benefit of both the program and the new residents.

Reviewer Expertise:

NA

No decision status is available

# Reviewer report: Reviewer response for version 1

**DOI:** 10.21956/mep.20019.r30547

**Published:** 2020-05-11

**Authors:** P Ravi Shankar

**Affiliations:** 1 American International Medical University

This review has been migrated. The reviewer awarded 5 stars out of 5 I enjoyed reading this well-written manuscript written mainly by alumni and members of the first batch of residents at the program. I could identify with many of the issues mentioned. During my undergraduate education in Kerala, India we did not have any postgraduates (residents) at our medical school and so we got undivided attention from our faculty and the opportunity to play an important role in patient management and to carry out procedures. We were also the inaugural batch during a health professions education fellowship and we had the undivided attention of our faculty but did miss the guidance of senior fellows and alumni. The methodology of the study has been well described. I would be interested in knowing more about the quarterly Voice in the program sessions. The lessons learned will be of interest to new residency and even new undergraduate medical programs in other settings.

Reviewer Expertise:

NA

No decision status is available

# Reviewer report: Reviewer response for version 1

**DOI:** 10.21956/mep.20019.r30545

**Published:** 2020-05-08

**Authors:** Trudie Roberts

**Affiliations:** 1 Leeds Institute of Medical Education

This review has been migrated. The reviewer awarded 5 stars out of 5 I started to read this paper with no expectations that it would be useful except in a US environment. I'm very glad I persisted in reading it as I was completely mistaken. This contribution is well written and of interest to any one setting up a postgraduate training programme. Some of the themes reported made me think of areas I might not have considered seriously eg the absence of senior residents and some themes re-confirmed the need for communication and faculty development. Thank you for an interesting and worthwhile contribution

Reviewer Expertise:

NA

No decision status is available
